# Effect of Methyl Gallate on 1-Nitropyrene-Induced Keratinocyte Toxicity in a Human and Canine Skin Model

**DOI:** 10.4014/jmb.2206.06004

**Published:** 2022-06-30

**Authors:** Woo Jin Lee, Min Jeong Kim, Hyun-Wook Choi, Jeong Jae Lee, Sung Keun Jung

**Affiliations:** 1 School of Food Science and Biotechnology, Kyungpook National University, Daegu 41566, Republic of Korea; 2 Department of Functional Food and Biotechnology, Jeonju University, Jeonju 55069, Republic of Korea; 3 Institute of Agricultural Science and Technology, Kyungpook National University, Daegu 41566, Republic of Korea; 4 Tailored Food Technology, Kyungpook National University, Daegu 41566, Republic of Korea

**Keywords:** Apoptosis, canine skin, HaCaT cells, methyl gallate, 1-nitropyrene, skin toxicity

## Abstract

The skin, which is the largest organ of the human body, is in direct contact with pollutants in the surrounding atmosphere. Meanwhile, 1-nitropyrene (1-NP), the most abundant nitro-polycyclic aromatic hydrocarbon found in particulate matter, is known to have carcinogenic effects; however, studies on its toxicity in human and canine skin are still needed. In this study, we investigated 1-NP-induced apoptosis and inflammatory pathways in HaCaT cells. In addition, we also measured the cytoprotective effect of methyl gallate (MG), which is widely distributed in medicinal and edible plants and is well known for its anti-inflammatory and antioxidant properties. MG inhibited 1-NP-induced cell death and apoptosis pathways, including the cleavage of PARP and activation of caspase-3, -7, and -9. MG also suppressed 1-NP-induced COX-2 expression and phosphorylation of mitogen-activated protein kinases (MAPKs) and MAPK kinases (MAPKKs). Our findings suggest that 1-NP induces skin toxicity in human and canine through apoptosis and inflammatory responses, and moreover, that this can be prevented by treatment with MG.

## Introduction

According to the World Health Organization (WHO), indoor and outdoor environmental pollution is considered the greatest health risk to human health, and the death rate from related diseases is increasing annually [1]. Among environmental pollutants, particulate matter (PM) is considered to be a severe toxicant and is carcinogenic to humans. In addition, companion animals, such as dogs and cats, are susceptible to various air pollution sources [2, 3]. The skin is an interface between the atmosphere and body and is primarily exposed to substances that are harmful to the environment [4]. Organic compounds such as PM-derived polycyclic aromatic hydrocarbons (PAHs) and nitro-PAHs are lipophilic and can easily pass through the skin and cause skin toxicity [5]. Among them, 1-nitropyrene (1-NP), which is a nitro-PAH that is also the most abundant compound among PM pollutants, is mainly found in diesel exhaust particles (DEPs) [6]. There is increasing evidence that 1-NP can be detected not only in DEPs but also in ash, household heater emissions, cigarette smoke, and urban dust [7]. However, no previous studies have evaluated the harmful effects of 1-NP on human or canine skin. Moreover, previous studies have reported that the keratinocyte-derived immune reactions are similar mechanisms in both human and canine [8-11]. Therefore, we investigated the effects of 1-NP treatment on the keratinocytes in human and canine.

In the skin, there is evidence that PM exposure plays a critical role in the pathogenesis of various skin diseases caused by apoptosis and inflammation [12, 13]. Apoptosis, also known as programmed cell death, is morphologically distinct from other types of cell death and is the process by which cells stop growing and dividing, without releasing their contents to the external environment, resulting in the controlled death of cells [14]. Apoptosis can be triggered by numerous stimuli and signaling pathways, including oxidative stress, cytokines, hormones, cell-cell interactions, drugs, and chemotherapeutic agents [12]. Overexpression of COX- 2 is frequently observed in acute or chronic inflammatory or cancerous tissue [15, 16]. As COX-2-specific inhibitors appear to be useful alternatives for the treatment of osteoarthritis and rheumatoid arthritis with high complication rates, the role of COX-2 in inflammation is clear and COX-2 inhibition is used as a therapeutic strategy against inflammation [17, 18].

Polyphenols are secondary metabolites found in plants and are abundant in the diet [19]. They are reported to exhibit health benefits through antioxidant, anti-inflammatory, and immunomodulatory activities [20]. Methyl gallate, a derivative of gallic acid, is a ubiquitous polyphenol that is widely distributed in medicinal and edible plants [21]. Several studies have shown that MG is effective against colitis [22], diuresis [23], kidney damage [24], and DNA damage [25] caused by oxidative stress. However, there have been no studies on the effect of MG in preventing 1-NP-induced skin toxicity. Therefore, in this study we also explored the cytotoxic signaling pathway expressed when HaCaT cells were treated with 1-NP, and evaluated the anti-apoptotic and anti-inflammatory effects of MG.

## Materials and Methods

### Materials and Reagents

Dulbecco’s modified Eagle’s medium (DMEM), fetal bovine serum (FBS), and antibiotics (penicillin/streptomycin solution) were purchased from Thermo Fisher Scientific, Inc. (USA). 1-NP was obtained from TCI Chemicals (Japan). MG was obtained from Sigma-Aldrich (USA).

### Cell Culture

The immortalized human keratinocyte cell line (HaCaT) was cultured in DMEM supplemented with 10% FBS and antibiotics. HaCaT cells were maintained at 37°C and 5% CO_2_ in a humidified incubator (Thermo Fisher Scientific).

### Cell Viability Assays

HaCaT cells were seeded at a density of 2 × 10^5^ cells/ml in a 96-well plate at a volume of 100 μl. When the HaCaT cells reached 80% confluence, the medium of the 96-well plate was changed to serum-free medium. After 18–24 h, HaCaT cells were treated with various concentrations of 1-NP or pretreated with MG for 1 h before 1-NP treatment. After 1 day, 10 μl of MTT solution was added to each well, and DMSO was added after 2 h to dissolve the formazan. After 1 h, the formazan was completely dissolved and the absorbance at 595 nm was measured using a microplate reader to determine cell viability (Bio-Rad, USA).

### Cell Morphological Changes

In a 12-well plate, HaCaT cells (2 × 10^5^ cells/ml) were seeded at a volume of 1 ml. After 1 day, the cells reached 80% confluence. The medium was then changed to serum-free medium. After 18–24 h, cells were treated with specific concentrations of 1-NP or pretreated with MG for 1 h before 1-NP treatment. Morphological alterations were examined using a microscope (Leica Microsystems, Germany).

### Western Blot Assays

HaCaT cells (2 × 10^5^ cells/ml) were seeded in 100-mm cell culture dishes. When the cells reached 80–90%confluency, the medium was changed to serum-free medium. Cells were treated with specific concentrations of MG 1 h before stimulation with 1-NP (5 μM) and incubated for various periods. Cells on the plates were placed on ice, the medium was removed, and the plates were washed twice with cold phosphate-buffered saline (PBS). Intracellular proteins were obtained by lysing cells with lysis buffer (Cell Signaling Technologies, USA) containing a protease and phosphatase inhibitor cocktail (Thermo Fisher Scientific Inc.) for 30 min with vortexing every 10 min. The protein content of the cell lysate was determined using a DC Protein Assay Kit (Bio-Rad Laboratories, Inc.) according to the manufacturer’s instructions. Proteins were electrically separated according to mass on an SDS-PAGE gel and transferred to a PVDF membrane at 4°C for 1.5 h (Millipore, USA). The protein-transferred membranes were shaken in Tris-buffered saline containing 1% Tween-20 (TBST) at room temperature. The protein-membranes were then shaken with corresponding primary antibodies overnight at 4°C. After washing thrice with TBST, the membranes were incubated with horseradish peroxidase (HRP) secondary antibodies (Thermo Fisher Scientific Inc.) at room temperature for 1 h. After washing the membrane three times with TBST, a chemiluminescence detection kit (ATTO, Japan) was used to observe the protein bands using Gene Gnome XRQ NPC (Syngene, UK).

### Statistical Analysis

Where appropriate, results are expressed as mean ± standard error of the mean (SEM). Each experiment consisted of at least three replications. One-way analysis of variance and Student’s *t*-test were used for statistical analyses. *p* < 0.05 was considered statistically significant.

## Results

### Methyl Gallate Prevents 1-Nitropyrene-Induced Cell Death in HaCaT Cells

**Fig. 1:**
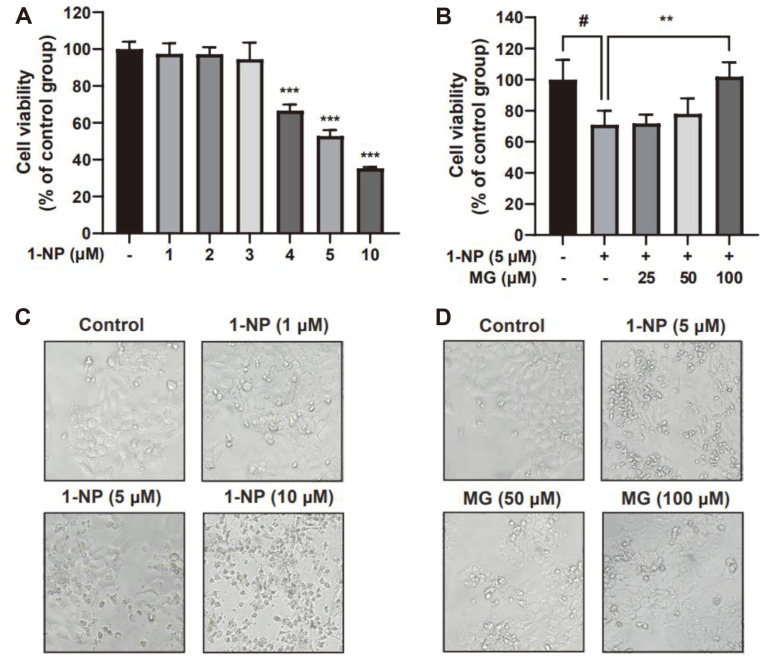
Methyl gallate (MG) inhibits 1-nitropyrene (1-NP)-induced HaCaT cell death. (**A**) Cytotoxicity of 1-NP in HaCaT cells. HaCaT cells were incubated with 1-NP (1-10 μM) for 24 h. **p < 0.01 and ***p < 0.001 between the control groups and the group exposed to 1-NP alone. Data are presented as the mean ± SD of three independent experiments. (**B**) MG inhibits 1-NP-induced toxicity in HaCaT cells. HaCaT cells were treated with MG (25, 50, and 100 μM) for 1 h and then exposed to 1-NP (5 μM) for 24 h. The viability of HaCaT cells was measured by MTT assay. #*p* < 0.05 between the control group and the group exposed to 1-NP alone; **p < 0.01 between the 1-NP + MG groups and the group exposed to 1-NP alone. Data are presented as the mean ± SD of three independent experiments. (**C**) Morphological changes of 1-NP-treated HaCaT cells. HaCaT cells were observed under a microscope after 1-NP (1, 5, and 10 μM) stimulation for 24 h. (**D**) MG alleviates 1-NPinduced morphological changes. HaCaT cells were pretreated with MG (50 and 100 μM) for 1 h and then exposed to 1-NP (5 μM) for 24 h.

The skin is the first line of defense against exposure to external contaminants. First, we evaluated whether 1-NP had cytotoxic effects on HaCaT cells. The cells were exposed to various concentrations of 1-NP (1, 2, 3, 4, 5, and 10 μM) for 24 h, and cell viability was determined using the MTT assay. Treatment with 1-10 μM of 1-NP significantly reduced the viability of HaCaT cells (Fig. 1A). Since 5 μM 1-NP induced approximately 50% cell death, we used this concentration in all subsequent in vitro experiments. Next, we confirmed the effects of MG on 1-NP-induced cytotoxicity in HaCaT cells using the MTT assay. As shown in Fig. 1B, MG significantly inhibited 1-NP-induced death in HaCaT cells. As shown in Fig. 1A and 1B, cell morphology was observed under a microscope. It was confirmed that as the 1-NP concentration increased, HaCaT cell death also increased (Fig. 1C). Microscopy revealed that MG prevented cell death in HaCaT cells treated with 5 μM 1-NP (Fig. 1D).

### Methyl Gallate Ameliorates 1-Nitropyrene-Induced Activation of the Apoptotic Pathway in HaCaT Cells

**Fig. 2:**
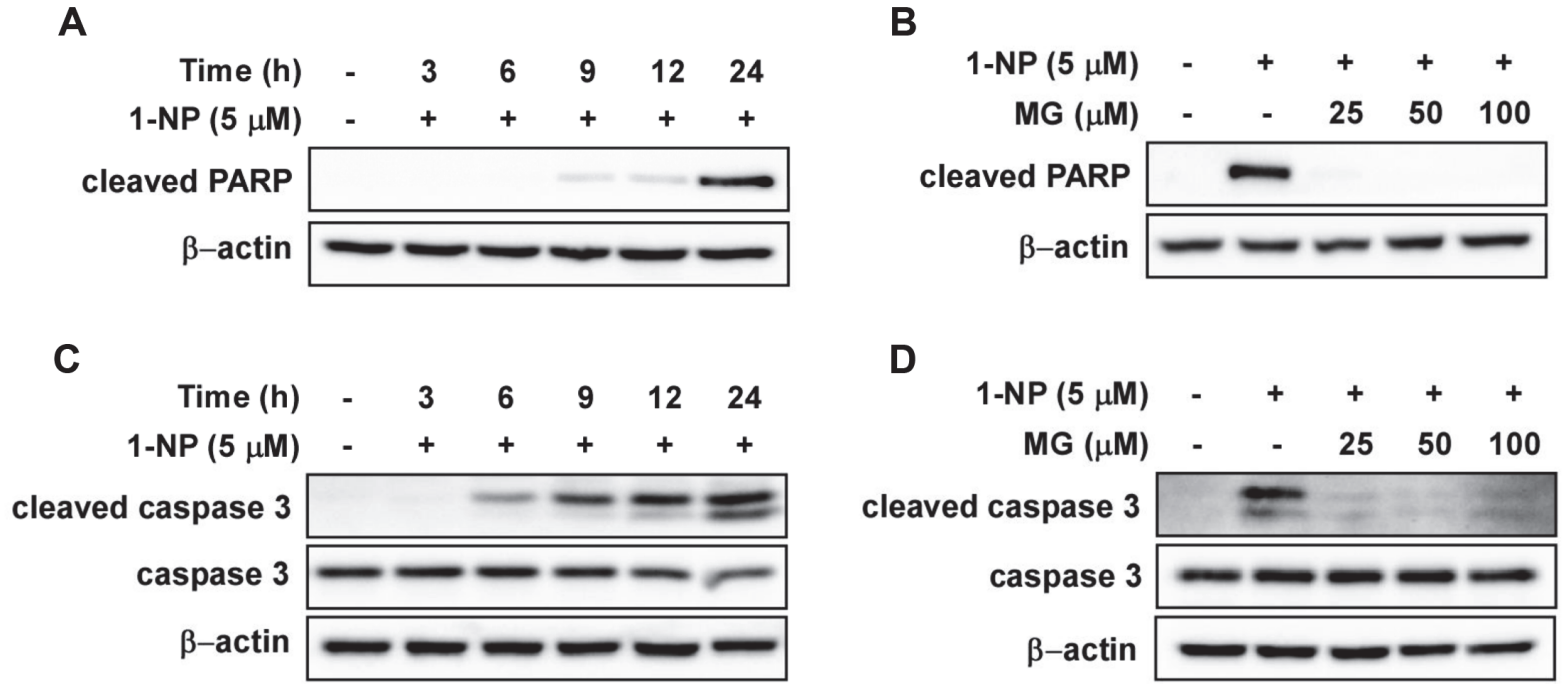
Methyl gallate (MG) inhibits 1-nitropyrene (1-NP)-induced apoptotic protein cleavage in HaCaT cells. (**A**) The expression levels of cleaved PARP were detected by western blotting. At specific times, HaCaT cells were harvested after 1-NP (5 μM) treatment. (**B**) The effect of MG (25, 50, and 100 μM) on the 1-NP-induced expression of cleaved- PARP in HaCaT cells. (**C**) The expression levels of cleaved caspase-3 were detected by western blotting. At given times, HaCaT cells were harvested after 1-NP (5 μM) treatment. (**D**) The effect of MG (25, 50, and 100 μM) on the 1-NP-induced expression of cleaved caspase-3 in HaCaT cells.

The type of cell death caused by 1-NP treatment of HaCaT cells needs to be confirmed. Since PARP cleavage is the most important feature of apoptotic cell death [26], we assessed whether treatment with 1-NP caused the cleavage of PARP. Western blot analysis showed that cleaved PARP expression was observed 9 h after 1-NP treatment and reached a maximum at 24 h (Fig. 2A). The effect of MG on 1-NP-induced cleavage of PARP was measured 24 h after 1-NP treatment, and MG was found to completely inhibit the generation of cleaved PARP (Fig. 2B). The activation of caspase-3 is an important factor in apoptosis because it causes PARP fragmentation [27]. Having confirmed the fragmentation of PARP, we investigated whether 1-NP activated caspase-3. After HaCaT cells were treated with 1-NP, caspase-3 cleavage increased dramatically over time (Fig. 2C). MG completely inhibited 1-NP-induced caspase 3-cleavage after 24 h (Fig. 2D).

### Methyl Gallate Regulated the Activation of the Apoptosis Pathway by 1-Nitropyrene Treatment in HaCaT Cells

**Fig. 3:**
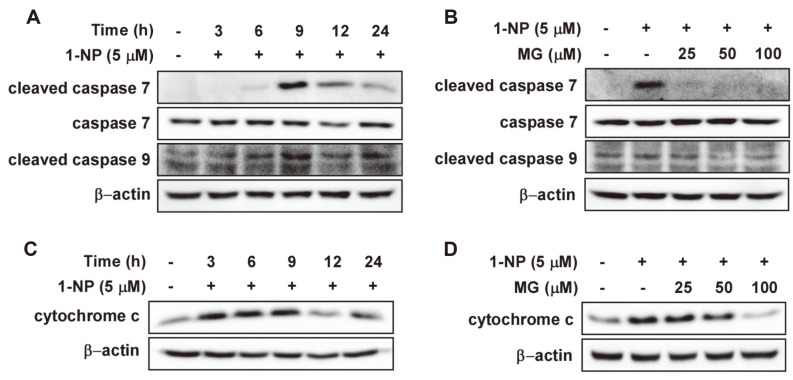
Methyl gallate (MG) prevented the 1-nitropyrene (1-NP)-induced apoptotic pathway in HaCaT cells. (**A**) The expression of cleaved caspase-7 and -9 proteins was determined by western blotting at given times after 1-NP (5 μM) treatment in HaCaT cells. (**B**) The levels of cleaved caspase-7 and -9 proteins were measured by western blotting. HaCaT cells were pretreated with MG (25, 50, and 100 μM) for 1 h and then exposed to 1-NP (5 μM) for 9 h. (**C**) The protein expression of cytochrome C was detected by western blotting. HaCaT cells were treated with 1-NP (5 μM) for various times. (**D**) The effects of MG on the 1-NP-induced expression of cytochrome C. HaCaT cells were pretreated with MG (25, 50, and 100 μM) and then treated with 1-NP (5 μM) for 9 h.

Caspase-7 and -9, which are upstream of caspase-3 activation, are intrinsic apoptosis signaling factors; therefore, we determined whether they are induced by 1-NP [28]. As a result of 1-NP treatment of HaCaT cells, it was confirmed by western blotting that the activation of caspase-9 and -7 occurred at 9 h (Fig. 3A). Additionally, MG significantly prevented the 1-NP-induced activation of caspase-7 and -9 in HaCaT cells (Fig. 3B). In the intrinsic apoptotic pathway, mitochondrial cytochrome C is released, which contributes to caspase activation [29]. Compared with the control group, cytochrome C levels increased markedly after 3 h (Fig. 3C). MG suppressed 1-NP-induced cytochrome C release in a concentration-dependent manner after 3 h (Fig. 3D).

### Methyl Gallate Suppressed 1-Nitropyrene-Induced COX-2 Expression and Phosphorylation of MAPKs in HaCaT Cells

**Fig. 4:**
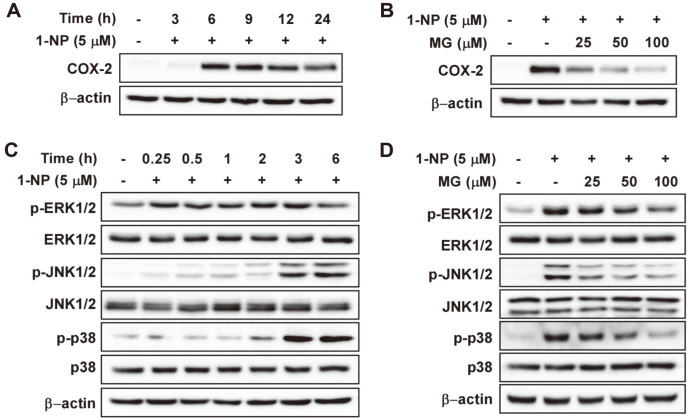
The effect of methyl gallate (MG) on the 1-nitropyrene (1-NP)-induced MAPK/COX-2 pathway. (**A**) 1-NP activates the expression levels of COX-2 proteins. HaCaT cells were treated with 1-NP (5 μM) for specific times. (**B**) MG inhibits the 1-NP-induced COX-2 expression in HaCaT cells. HaCaT cells were pretreated with MG (25, 50, and 100 μM) for 1 h and then exposed to 1-NP (5 M) for 6 h. (**C**) 1-NP activates phosphorylation of MAPKs in HaCaT cells. HaCaT cells were treated with 1-NP (5 μM) for specific times. (**D**) MG suppresses 1-NP-induced phosphorylation of MAPK in HaCaT cells. Cells were pretreated with MG for 1 h, treated with 1-NP, and harvested after 3 h. Phosphorylation and expression levels were detected by western blot analysis.

Persistent or acute skin inflammatory reactions can cause skin diseases including rosacea, psoriasis, atopic dermatitis, and skin cancer [30]. Cyclooxygenase (COX)-2 is a major marker of skin inflammation [31]. Accordingly, we investigated whether 1-NP induced COX-2 expression in HaCaT cells using western blotting. As shown in Fig. 4A, the 1-NP-induced increase in COX-2 expression was maximized at 6 h. MG suppressed the 1-NP-induced COX-2 expression in HaCaT (Fig. 4B). The expression of COX-2 is induced through various signaling processes, but all these processes eventually converge to the activation of mitogen-activated protein kinases (MAPKs) [32]. Based on the increase in COX-2 after 1-NP treatment, we evaluated the phosphorylation of MAPKs induced by 1-NP treatment. Significant extracellular signal-regulated kinase (ERK) phosphorylation occurred after 15 min of 1-NP treatment. The phosphorylation of c-Jun N terminal kinases (JNK)1/2 and p38 increased with time and peaked after 3 h (Fig. 4C). MG significantly inhibited 1-NP-induced phosphorylation of ERK1/2, JNK1/2, and p38 in a dose-dependent manner, while total ERK1/2, JNK1/2, and p38 protein levels were unaffected (Fig. 4D).

### Methyl Gallate Ameliorates 1-Nitropyrene-Induced Phosphorylation of MAPKKs in HaCaT Cells

**Fig. 5:**
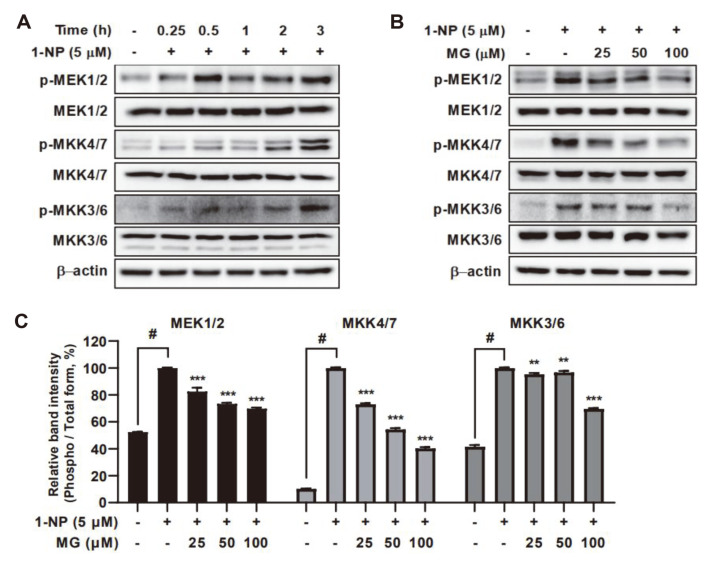
The effects of methyl gallate (MG) on 1-nitropyrene (1-NP)-induced MAPKKs phosphorylation in HaCaT cells. (**A**) The protein expression levels of phosphorylation of MAPKKs were detected using western blotting. HaCaT cells were stimulated with 1-NP (5 μM) at various times. (**B**) MG inhibits the 1-NP-induced activation of MAPKKs in HaCaT cells. HaCaT cells were pretreated with MG (25, 50, and 100 μM) for 1 h and then incubated in the presence of 1-NP (5 μM) for 3 h. The protein levels of total MAPKKs and phosphorylation of MAPKKs were measured by western blotting. #*p* < 0.05 between the control groups and the group exposed to 1-NP alone; **p < 0.01 and ***p < 0.001 between the 1-NP and MG groups and the group exposed to 1-NP alone.

MAPKK, known as an upregulator of MAPKs, is a kinase enzyme that activates and phosphorylates MAPK proteins [33]. Thus, we evaluated whether 1-NP induced the phosphorylation of MAPKKs in HaCaT cells using western blotting. Phosphorylation of mitogen-activated protein kinase (MEK)1/2 was strongly induced in the early stages, whereas phosphorylation of mitogen-activated protein kinase (MKK)4/7 and MKK3/6 was gradually induced and reached a maximum at 3 h (Fig. 5A). However, MG suppressed the 1-NP-induced phosphorylation of MEK 1/2, MKK4/7, and MKK3/6 in a concentration-dependent manner (Figs. 5B and 5C).

## Discussion

Global air pollution has become a major cause of health deterioration [34]. The skin is the first line of defense against fine dust and is the largest organ interfacing directly with the atmosphere [35]. DEPs are a major component of PM pollutants and consist of numerous PAHs and nitro-PAHs [36]. 1-NP is the most abundant nitro-PAH among 200 types of nitro-PAH in fine dust, and it is known that 1-NP is 700 times more abundant than B[a]P, a carcinogen, in SRM 2975, the standard material for DEP [37]. Accumulated evidence suggests that 1-NP is a molecular marker of DEP and has potential as a biomarker for detecting DEP exposure through metabolite quantification [38-40]. Thus, 1-NP is a chemical indicator of DEP. In this study, we identified the mechanism by which 1-NP causes skin toxicity and confirmed its protective effect. 1-NP can induce skin inflammation through the MAPK pathway and has been shown to induce mitochondrial apoptosis via the MAPK pathway. Furthermore, we showed for the first time that MG significantly ameliorated 1-NP-induced apoptosis and inflammatory responses in HaCaT cells.

Cell death is triggered by actions affecting the cell and can be broadly classified into necrosis, autophagy, and apoptosis [41]. Triggering apoptosis is a potential therapeutic strategy in cancer, as it is a form of programmed cell death that efficiently removes cells with DNA damage or damage during development [42]. We confirmed cell death caused by 24-h 1-NP treatment in HaCaT cells. After that, we sequentially confirmed the fragments of PARP as well as caspases-3, -7, and -9 by western blotting, indicating caspase-related mitochondria-induced apoptosis. Since PARP-1 functions to repair and replicate DNA damage, cleavage of PARP, which is known to be induced by caspase-3 activation, inhibits DNA repair and replication, thereby increasing DNA damage [43]. The cleavage of PARP by 1-NP has been confirmed in previous studies in macrophages (Hepa1c1c7 and Beas-2B cells) [36, 44, 45]. Additionally, MG affected overall intrinsic apoptosis and prevented 1-NP-induced apoptosis.

Excessive COX-2 expression has long been known to play a pivotal role in the pathogenesis of inflammation [46]. We confirmed that COX-2 expression increased non-ideally as a result of 1-NP treatment of HaCaT cells, and 1-NP may induce an inflammatory response in the skin. We also evaluated the influence of MAPKs, which are known to be a significant factor in COX-2 expression, through western blotting [47]. 1-NP also increased the phosphorylation of MAPK members ERK1/2, JNK1/2, and p38. In Hepa1c1c7 cells treated with 1-NP, the same upregulation of MAPKs was also shown [37]. MAPKKs independently and directly phosphorylate MEK1/2 on ERK1/2, MKK4/7 on JNK1/2, and MKK3/6 on p38 [48]. We confirmed that 1-NP significantly increased the phosphorylation of MAPKKs upstream of MAPK. Based on this, it is evident that 1-NP can induce an inflammatory response in skin cells by passing the MAPK pathway and expressing COX-2, an inflammatory factor. Activation of MAPKs, including c-Jun N-terminal kinase (JNK) and p38, is known to affect apoptosis [49]. MAPKs affect numerous cellular stimuli, and the responses are significantly involved in inflammation and apoptosis [50]. We further confirmed that when cells were pretreated with MG for 1 h before 1-NP treatment, the inflammatory response could be prevented by inhibiting the activation of the MAPK/COX-2 pathway caused by 1-NP treatment.

Regarding skin damage, numerous studies have been performed on photoaging, atopy, or physical tissue damage, such as friction and burns caused by UV irradiation. However, studies on the action of the major compounds in fine dust are lacking, and further research is needed. This study is the first to elucidate the mechanism of skin toxicity of 1-NP, which is the most abundant nitro-PAH in fine dust, and the beneficial effects of MG.

In this study, we found that 1-NP, an abundant compound in environmental pollutants, induces intrinsic apoptosis through the mitochondrial caspase signaling pathway and inflammation via COX-2 expression generated by the MAPK pathway in HaCaT cells. Additionally, we observed that MG prevented the 1-NP-induced intrinsic apoptosis pathway and COX-2 expression through the inhibition of MAPKs/MAPKKs. Overall, these results reveal that 1-NP is a harmful compound that induces skin toxicity in human and canine, and MG could be a promising anti-apoptosis and anti-inflammatory agent of 1-NP-mediated skin toxicity in both cases.
